# Lithium Ion Battery Anode Aging Mechanisms

**DOI:** 10.3390/ma6041310

**Published:** 2013-03-27

**Authors:** Victor Agubra, Jeffrey Fergus

**Affiliations:** Materials Research and Education Center, Auburn University, 275 Wilmore Laboratories Auburn, AL 36849, USA; E-Mail: vaa0002@auburn.edu

**Keywords:** Li-ion battery anode materials, Li-ion conduction, composite electrodes

## Abstract

Degradation mechanisms such as lithium plating, growth of the passivated surface film layer on the electrodes and loss of both recyclable lithium ions and electrode material adversely affect the longevity of the lithium ion battery. The anode electrode is very vulnerable to these degradation mechanisms. In this paper, the most common aging mechanisms occurring at the anode during the operation of the lithium battery, as well as some approaches for minimizing the degradation are reviewed.

## 1. Introduction

The high energy/ power density, and excellent cycle life of the lithium ion battery have positioned it as the preferred portable energy source for consumer appliances and in the automotive industry. The performance of the battery depends on the development of materials for the various components of the lithium ion battery [1,2,3]. The degradation of these components during battery operation adversely affects the energy delivery of the lithium ion battery.

The various battery components undergo different aging mechanisms; the binder and electrolyte decompose, the current collector corrodes, the separator melts and corrodes, and the cathode undergoes structural disorder and metal dissolution. 

The anode undergoes a multitude of aging mechanisms that degrade the electrochemical performance of the lithium ion battery. The most commonly used anode materials include carbon-based compounds and lithium-alloys. The microstructure, texture, crystallinity and morphology of the anode material directly influence its performance [4].

By design, the anode electrode has a large geometry dimension compared to the cathode electrode so as to prevent edge lithium plating at the anode ends. Also, the anode electrode generally has excess anode capacity compared to the cathode so that the battery can deliver high energy density [5]. A reduced anode capacity will polarize the anode to a potential close to lithium deposition potentials. However, higher surface area of the electrode is preferred to active surface area, since a higher surface allows short diffusion paths for lithium ions between the graphite particles; this facilitates fast charge and discharge rate and improves the capacity of the battery. Decrease particles size tends to increase the specific surface area from BET (Brunauer Emmett and Teller) method and the irreversible capacity loss increases. On the other hand, irreversible capacity decreases as the active specific area of the electrode increases. The intercalation of lithium ions into the graphite sheets at various stages, e.g., Li*_x_*C_6_, Li*_x_*C_12_, during the charging cycles to provide a nominal theoretical capacity of the carbon based anode of about 372 mAh/g. Additives, such as B, N and P, have been used to enhance this capacity.

Nevertheless, the anode has been associated with many aging mechanisms in the lithium ion battery. The focus of this paper is to elucidate the various aging mechanisms occurring at the anode of the lithium ion battery. Although the main focus will be on the aging mechanisms, a brief analysis of various treatment measures adopted to mitigate these aging mechanisms on the anode will be discussed.

## 2. Formation of Passivated Surface Layer 

**Figure 1:**
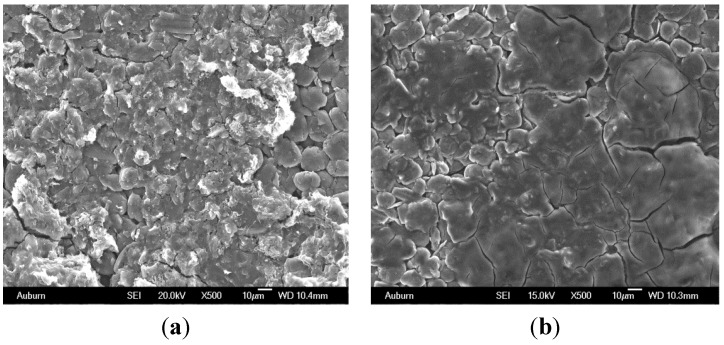
Growth of the passivation layer on the anode resulting from electrolyte decomposition. (**a**) Surface film agglomerates; (**b**) Surface film forms a “mat” on the carbon particle surface area.

Graphite is one of the common anode materials for lithium ion batteries operating in organic electrolytes, such as LiPF_6_, with co-solvents like ethylene carbonate (EC), dimethyl carbonate (DMC), diethyl carbonate (DEC), methyl ethyl carbonate (EMC)). The reaction of the anode with the electrolyte solution in the formation stage results in the formation of species such as ROCO_2_Li and CO_2_OLi, on the anode surface. The layer formed by these species is referred to as the solid electrolyte interphase (SEI). The ROCO_2_Li can undergo reduction reaction with CO_2_ and traces of H_2_O in the electrolyte to form lithium carbonate [6] which further react with EC to form transesterification products such DMDOHC, EMDOHC and DECDOHC. In addition, anion contaminates, such as F^−^ from HF and PF_5_, readily react with lithium to form insoluble reaction products which are non-uniform, electronically insulating, and unstable on the surface of the graphite particles [7,8,9,10,11]. In addition, the dissolution of the cathode electrode metal from the lattice into the electrolyte due to the disproportionation of Mn^3+^ (into Mn^2+^ and Mn^4+^) by traces of hydrofluoric acid (HF) in the electrolyte, resulting in the deposition of cation contaminates, such as of Mn, Co and Fe, on the anode electrode surface [12],

At higher battery potentials, during the intercalation of lithium ions into the anode lattice structure, the graphite anode oxidizes. At this potential, electrolyte co-solvents, such as EC, which is highly reactive, react with the lithium ions and the reaction products quickly precipitate and grow on the anode surface [13,14]. The presence of these reaction products on the surface retards the intercalation kinetics of the carbon anode [15]. The surface layer grows in thickness as the decomposition reaction continues [16,17,18,19,20,21,22]. The layer thickness is established to be a function of operating cycles, regardless of the charging protocol (*i.e.*, pulse charging or DC charging) [23,24,25]. The layers become unstable and crack due to expansion and contraction of the graphite lattice during the insertion and de-insertion of the lithium ions [26,27,28]. This allows further surface reaction at these sites that may eventually isolate the graphite particles from the current collector. Figure 1 shows a typical surface film morphology and cracking of the layer (e.g., [26,27]). The surface crack formed on the surface does not typically travel to the carbon electrode [26]. The formation of this surface film layer is the predominate source of lithium ion loss in lithium ion battery during storage conditions [25]. It also leads to an increase in the charge transfer resistance, impedance, and clogs pores on the carbon anode electrode [29,30,31], which limits accessibility of lithium ions to the anode surface leading to an increase in irreversible capacity [32,33,34]. The growth of this surface layer on the anode electrode is prevalent in the electrolyte system with EC as the co-solvent compared to those with DEC or DMC as co-solvents [35,36,37,38].

## 3. Anode Impedance

**Figure 2:**
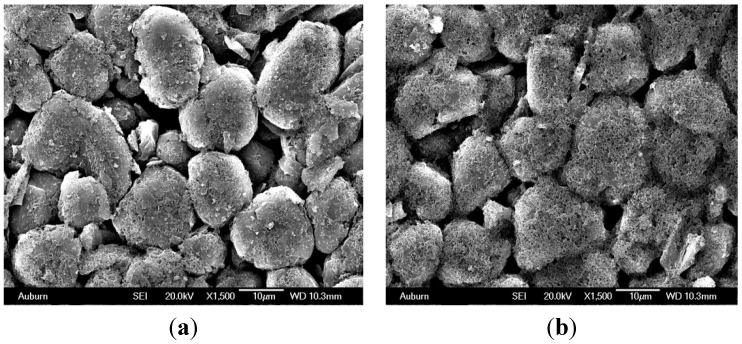
A typical SEM micrograph of surface film growth on the graphite particles. (**a**) Small precipitate of surface film on the carbon particles; (**b**) Precipitate thickness increases.

The growth of the passive surface layer on the anode creates resistance to lithium ion flow, which results in a rise in the charge transfer resistance and the impedance of the anode [39,40]. This increase in anode impedance is said to increase with charge rate, cycle number, temperature, and anode material particle size [41,42,43]. However, at low temperatures (10–30 °C) and low charge rate(C/20), the anode electrode contribution to the overall battery impedance is low. This is attributed to the small amount of the surface film formed on the electrode surface [44]. The low charge rate limits the amount of excess Li^+^ that is not intercalated into the electrode to react with the electrolyte [45,46]. A typical SEM micrograph of anode covered with products of electrolyte decomposition reaction products is shown in Figure 2. (e.g., [38,39,44,46,47]). Common surface reaction products formed on the anode surface include Li-alkyl carbonates, lithium carbonate species and fluorinated products. These products affect the intercalation and de-intercalation kinetics of the anode, and thus result in an increase in anode electrode impedance relative to the cathode [47,48,49].

**Figure 3:**
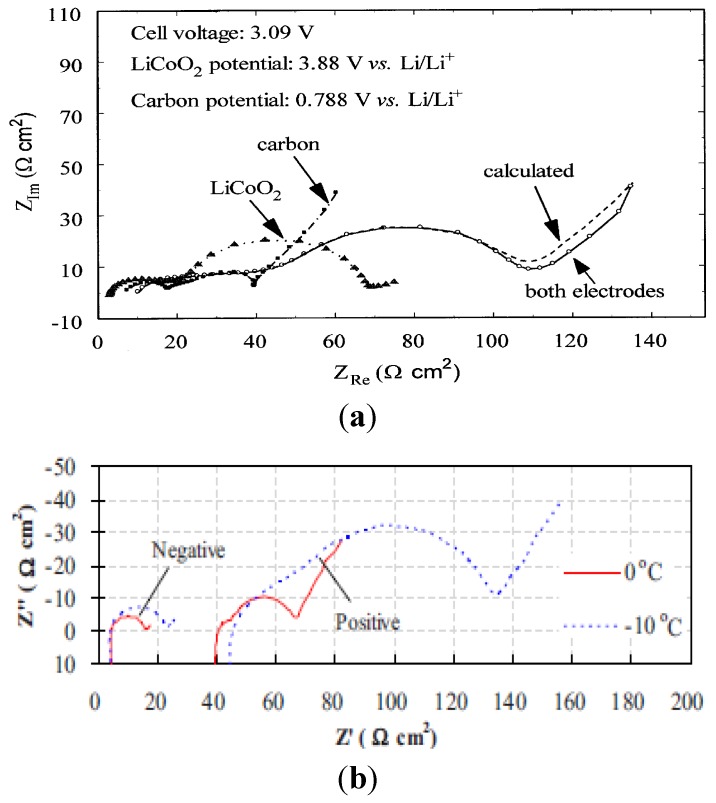
(**a**) Anode contributing less to total battery impedance (charge rate of C/2 at room temperature) [39]; (**b**) Anode contributing less to total battery impedance (graphite and LiNi_0.8_Co_0.15_Al_0.05_O_2_ electrodes: 0.5C charge rate at 40 °C) [45].

Comparing the impedance of the anode is difficult owing to the multitude of testing conditions and different anode materials used in various batteries. However, Figure 3a,b shows the impedances of the individual electrodes as well as the overall battery impedance. In these two cases, the anode contributes less to the overall battery impedance compared to the cathode. In these two cases, carbon is the anode material, while LiPF_6_ in EC+DMC is the electrolyte system and the operating conditions are low-to-medium temperature and low charge rate. The higher battery impedance exists at high operating temperature and charge rate, where the surface reactions are enhanced and a thicker surface film layer is formed on the electrode surface.

## 4. Degradation Due to the Loss of Recyclable Lithium Ions

The irreversible lithium ion loss is generally attributed to two phenomena, namely: (i) solid electrolyte interface (SEI) layer formation via electrolyte decomposition at the formation stage; (ii) side reaction of lithium ion with decomposed electrolyte compounds and water (e.g., 10–1500 ppm) in the electrolyte at the later stage of the battery operation [50].

The loss and/or consumption of recyclable lithium ions at the anode by the passive layer is a major cause of the reduction in the reversible capacity of the lithium ion battery [51,52]. As the layer grows, lithium is consumed in the reaction and the increased thickness inhibits Li^+^ transfer, thus the lithium ions must tunnel through the layer. This phenomenon is the main degradation mechanism in fully charged batteries at storage conditions [52,53,54,55], where the electronic insulating surface layer formed clog the pores and isolate graphite particles. The irreversible lithium ion loss is also a function of the specific area of the graphite particles, since an increase in area increases the volume of reaction products [56,57]. For a graphite anode with low specific area, the charge loss is low. The electrolyte additive, vinylene carbonate (VC) is one that increases the lithium ion loss rate at the anode for the Li/coke electrode during storage (ambient temperature conditions). Because it increases the rate of SEI formation reaction at ambient temperature conditions to increase the SEI thickness. However, its beneficial effect is seen at higher temperature (35–50 °C) and higher voltages >0.4 V for Li/coke, electrode as it slows down the side reaction rate and undergoes reduction and polymerization to form poly alkyl Li-carbonate species that suppress both solvent and salt anion reduction on the anode electrode.

Similarly, in batteries stored at voltages greater than 3.6V, electrolyte oxidation at the cathode can also induce surface reaction deposits that cover the active cathode electrode area. These covered areas are insulating, which could result in a non-homogeneous local current distribution in the cathode electrode.

## 5. Anode Degradation Due to Structural Changes 

**Figure 4:**
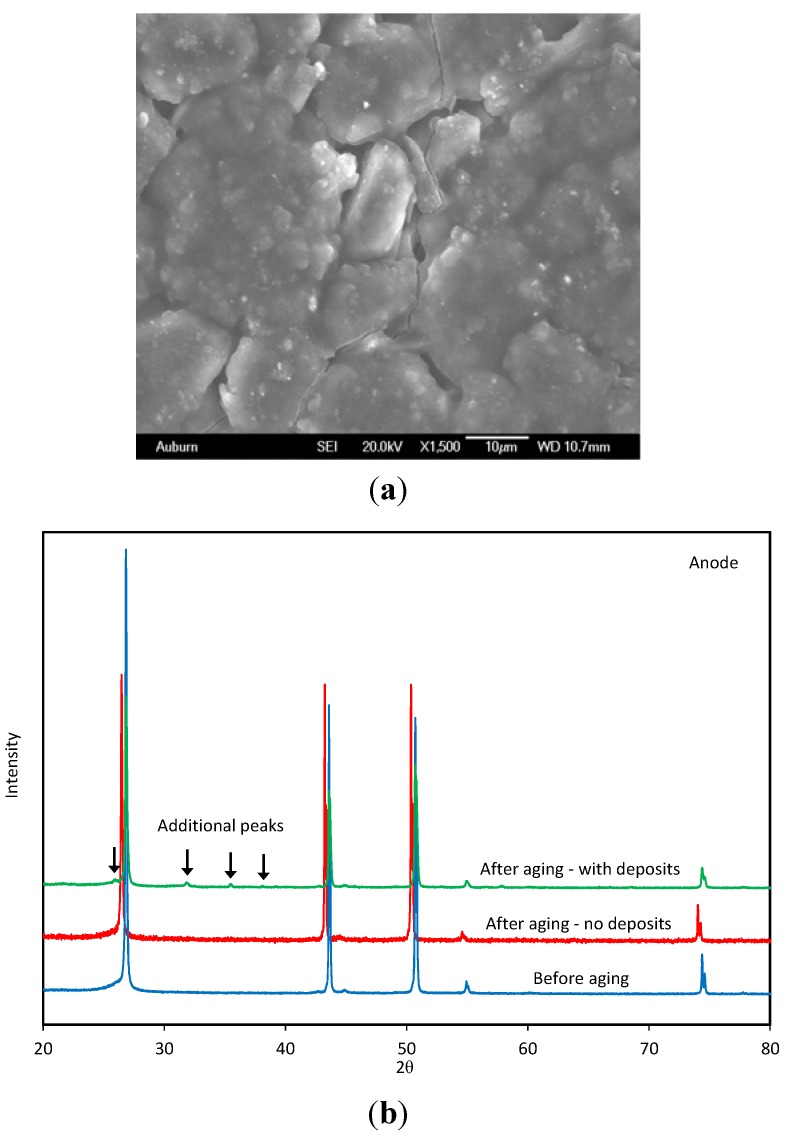
Structural changes on the anode electrode from degradation. (**a**) Surface cracks on the sufrcae of aged anode electrode; (**b**) XRD spectra of aged anode electrode showing change in cystal structure (new phases).

Anode materials, such as mesosphere pitch-based carbon (MSPBC) and vapor grown carbon fibers (VGCF), have high surface area morphologies that provide large discharge capacity and high charge rate performance [4]. During battery degradation, the ordered and radial structures of the carbon electrode may become less ordered, but this structural change is not the main contributor to battery degradation [58]. Degradation can be either in the form of the lithium plating or the formation of the surface film (e.g., new XRD peaks as in Figure 4). Neither the particles size nor the lattice parameter change significantly with these degradation processes [59]. 

Cycling the lithium ion batteries at high C-rate and high state of charge (SOC) induces mechanical strain on the graphite lattice of the anode electrode due the steep gradient of lithium ions, and thus lattice parameter, in the particle. This mechanical strain caused by the insertion and de-insertion of the lithium ions cracks, fissures and splits the graphite particles thus making these particles less oriented as compared to the original platelets [60]. Pressed graphite particles improve the ionic conductivity with a trade off in a decreased ohmic resistance and irreversible capacity loss [61]. The nature and orientation of the graphite particles influences the reversible capacity of the anode. For instance, less-oriented graphite particles have a low reversible capacity due to more difficult lithium intercalation kinetics and to the formation of new boundaries between crystallite at which irreversible lithium ions/electrolyte interaction can occur [62,63,64,65,66]. While flake-like graphite particles have higher gravimetric capacity at higher C-rate compared to spherical particles [67]. Although the crystal structure of the anode typically does not change with aging, a change in the rhombohederal/hexagonal content during battery operation has been reported. The increase in the hexagonal content during the first and third stage of lithium ions intercalation lowers Faradic efficiency, thereby decreasing the reversible capacity of the anode [68,69,70], so ideally a high ratio of rhombohederal/hexagonal content which gives a high reversible capacity is most desired.

## 6. Influence of Particle Size, Active Surface Area and Porosity 

**Figure 5:**
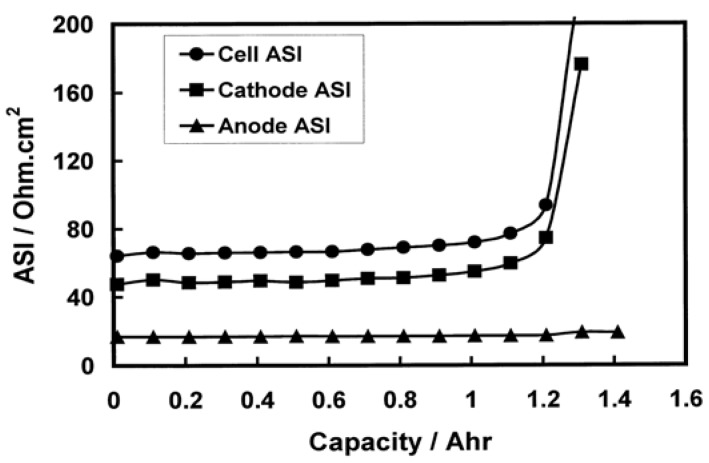
Effect of area specific impedance (ASI) on charge capacity [76].

**Figure 6:**
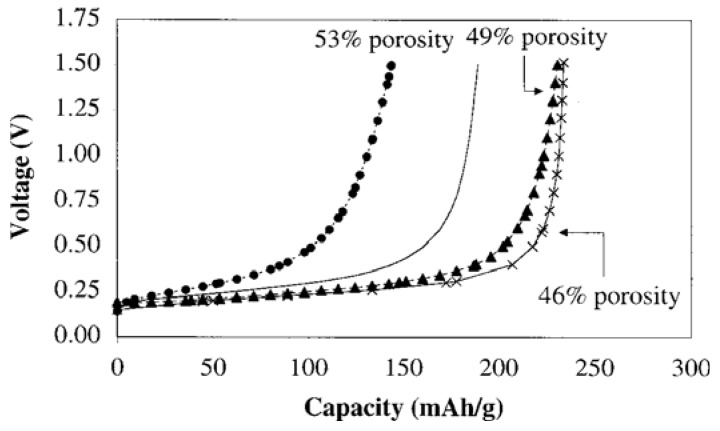
Effect of anode porosity on charge capacity for prismatic cell and cylindrical cell charge at 1C at 300 cycles [77].

The size of the graphite particles in the anode greatly influences the performance of the anode. Small particles contain short diffusion paths between the graphite particles, which facilitate fast charge and discharge rate [71]. Similarly, the larger surface area of the smaller graphite particles are prone to higher internal heat generation and lithium ion are consumed during the exothermic reaction at high temperatures greater than 60 °C [72] compared to larger particles size, this leads to an increase in the irreversible capacity of the graphite electrode [73,74,75]. The area specific impedance (ASI) of the graphite particles remains constant and does not vary much with capacity of the battery until the maximum capacity is reached [76] as shown in Figure 5. In the same vein, there has not been a direct correlation between the porosity of the graphite and the reversible capacity of the anode [77]. Figure 6 is a selected plot of graphite anode porosity data for prismatic and cylindrical (1.5 V) batteries cycled at 1C. An increase in porosity decreases the active surface area, reduces the electrical path into the graphite particles and reduces the accessibility of the lithium ions into the current collector. Although the pores will accommodate a large volume of electrolyte, they serve as a reaction point during the electrolyte decomposition process.

The density of the graphite anode has an effect on its ability to withstand degradation under strenuous battery operating conditions. A higher anode electrode particle density decreases the porosity and by extension, the active surface area of the electrode which reduces the electrode/electrolyte contact area. Therefore the denser the graphite material, the lower the irreversible capacity [78,79]. Furthermore, increased heat generation from a denser electrode material produces gaseous species [79] at temperatures greater than 120 °C from the thermal decomposition of the SEI layer. This implies that the thermal stability of the graphite anode is strongly dependent on the particle size of the graphite electrode.

## 7. Metallic Lithium Plating on the Anode

**Figure 7:**
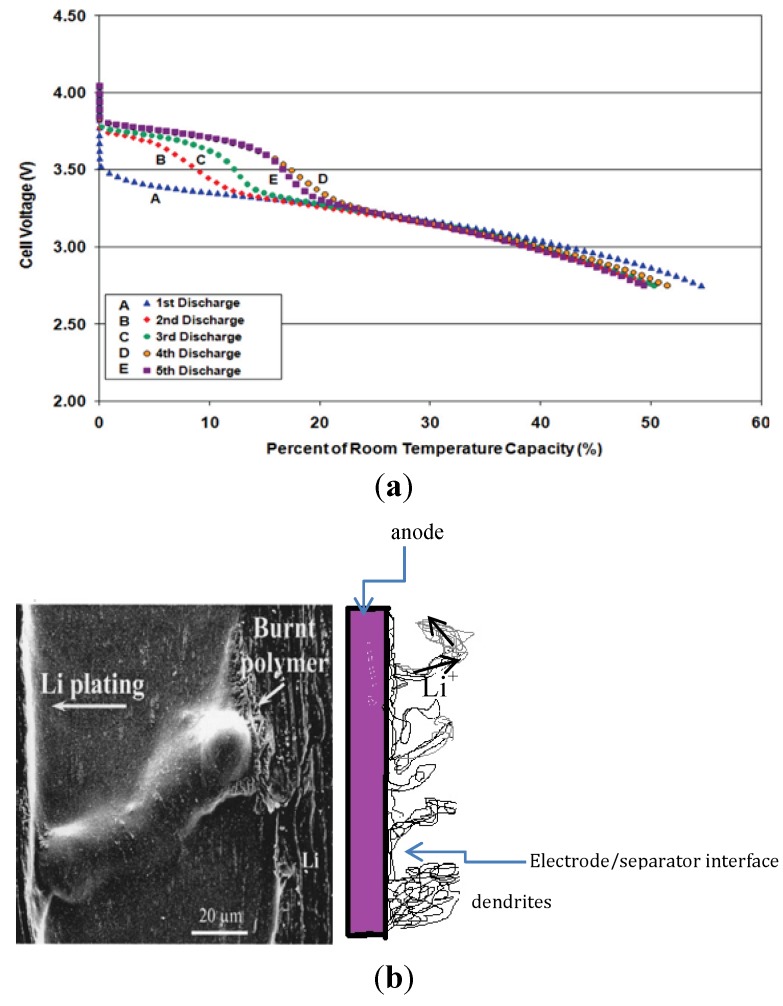
(**a**) Schematic current voltage over-potential caused by lithium plating [81]; (**b**) Lithium metal morphologies [82].

Its light weight, high voltage and high energy density once made lithium metal foil the preferred anode electrode for the lithium ion battery. However, its propensity to the formation of dendrites and moss made it unattractive. In the light if this, many more anode materials have been developed to replace the lithium metal foil as an anode material. Common anode materials currently used in lithium ion batteries include graphite, coke, hard carbon and lithium titanate. Among these, the unmodified graphite electrode is most susceptible to lithium plating because of the close proximity of its reversible potential to that of Li^+^/Li [80,81]. Lithium plating by itself is reversible, as the plated lithium oxidizes at potential of about 100mV, a potential much lower than that of lithium de-intercalation at the anode electrode, causing a voltage overshoot during the discharge cycle (over potential) as shown in Figure 7a. Well-ordered carbon and non-graphitizable carbon have gradually replaced lithium metal as the preferred anode material for the lithium ion battery because of their superior capacity, good cycleability, lower susceptibility to lithium plating, and low electrode potential relative to Li^+^/Li.

There are several factors that initiate the formation of metallic lithium on the surface of the anode electrode, some of these include: (1) the nature of the electrolyte (*i.e.*, electrolyte formulations with high EC content exhibit lithium plating); (2) the ratio between anode and cathode capacities (*i.e.*, low anode/capacity ratio will polarize the anode and promote lithium plating); (3) the operating temperature and the charge rate [*i.e.*, low temperature (−20 °C) and high charge rate] all influence plating on the anode [81,83]. These factors affect the anode kinetics and the lithium ion diffusion rate, such that lithium plates on the surface of the electrode rather than intercalating into the lattice of the carbon.

The formed metallic lithium deposits on the graphite anode are affected by the degree of random orientation of the particles in the crystal structure in the anode material and the non-uniformity of the current distribution which is a function of diffusion and current density [84,85]. The disorientation of the particles in the graphite electrode initiates inhomogeneity in the charge distribution on the anode electrode in the third and fourth intercalation stages and results in the formation of moss-like deposits and dendrites [82,86,87]. These moss-like deposits and dendrites grow as a function of the temperature and current density between the polymer separator and the anode. As the temperature and charge rate increases, the reaction rate also increases and metallic lithium is deposited on the graphite at overcharge. Dendrites can cause the separator to disconnect and become isolated from the electrolyte and in some instances pierce through the separator. The mat of dead lithium and dendrites can cause a short circuit and thermal runway in the battery (e.g., Figure 7b). The signature of lithium plating in batteries is usually manifested as a voltage plateau on the discharge voltage profile and low columbic efficiency [88]. 

The vulnerability of the anode electrode to degrade rapidly has prompted research to improve its stability. Several methods have been explored, including the inclusion of stabilizing compounds into the graphite matrix, formulations of dendrite and lithium plating suppression electrolyte systems. Elements such as Sn and carbon have been dispersed on the surface of the graphite to improve the electrochemical cycling properties of the anode electrode [89,90,91,92]. Sn on the surface of carbon anode reduces the SEI resistance and the overall electrode polarization at low temperature [93]. Also Sn-graphite anode increases passivation layer conductivity. While a carbon black coating suppresses the delithiation process in the inner structure of the graphite at elevated temperature and thus improves cycle life and capacity fade [94,95]. 

Another category of treatment on the surface of the anode electrode is the coating of the surface with additives like AD25, AsF_6_, VC and by thermal oxidation of the surface of the anode [95,96]. Thermal oxidation of graphite in air increases the surface area and fractional edge sites, which increases pores size and reduces particles size [97,98,99] and thus reduces the non-homogeneity of charge distribution that cause lithium plating. The additives AD25 and AsF_6_ stabilize the graphite at elevated temperature and suppress the formation of metallic lithium and reduction products of the LiPF_6_ [20,100,101]. Lastly, mechanical compression of the graphite particles during the electrode preparation process reduces the pore size thereby reducing the non-homogeneity of the charge distribution on the electrode and improving the reversible and irreversible capacities [101].

## 8. Conclusions

The anode of the lithium ion battery undergoes several degradation mechanisms during aging. Lithium plating is one aging mechanism which ends the life of a battery more rapidly due to the formation and growth of lithium dendrites. The decomposition of the electrolyte and subsequent formation of the film surface layer on the anode, cause an increase in the impedance and the consumption of recyclable lithium ions. These degradation mechanisms rarely affect the crystal structure of the anode electrode. The addition of various stabilizers, robust electrolyte systems, and temperature treatment are some of the methods that have been adopted to mitigate these aging effects on the electrode. However, further improvement is still needed to build a more robust anode that can deliver high energy density and good cycleability at various operating conditions. 
